# Whole Genome Sequencing and Comparative Analysis of *Bartonella bacilliformis* Strain INS, the Causative Agent of Carrion’s Disease

**DOI:** 10.1128/genomeA.00053-12

**Published:** 2013-02-14

**Authors:** D. Tarazona, C. Padilla, O. Cáceres, J. D. Montenegro, H. Bailón, G. Ventura, G. Mendoza, E. Anaya, H. Guio

**Affiliations:** a Laboratorio de Biotecnología y Biología Molecular, Instituto Nacional de Salud, Lima, Peru; b Laboratorio de Metaxénicas Bacterianas, Instituto Nacional de Salud, Lima, Peru; c Asociación Latinoamericana de Biotecnología (ALBIOTEC), Lima, Peru

## Abstract

*Bartonella bacilliformis* is the etiological agent of human bartonellosis, which is highly endemic to Peru. Here, we report the first genome that was sequenced and analyzed from an isolate of *B. bacilliformis* strain INS, which originally was isolated from the blood of an infected patient with an acute phase of Carrion’s disease from Jaén-Cajamarca, Peru.

## GENOME ANNOUNCEMENT

Carrion’s disease, caused by *Bartonella bacilliformis*, is commonly localized in the Andean region of South America, particularly in the valleys lining the western side of the Andes (1). Peru has reported rates of 242 per 100,000 inhabitants in the regions of Ancash, Cajamarca, and Amazonas (http://www.dge.gob.pe/).

Carrion’s disease has two clinical phases. During the acute phase, also called “Oroya’s fever,” *B. bacilliformis* invades red blood cells and produces severe hemolytic anemia (2). The chronic phase, also known as “verruga peruana,” is characterized by skin nodules and mulaire lesions, which usually bleed and lead to fibrosis (3). The differences in the manifestation of the disease during the two phases of bartonellosis suggest a complex interaction between *B. bacilliformis* and the human host that may involve a multitude of both bacterial and host proteins. Here, we report the whole-genome sequence of *B. bacilliformis* INS, which was isolated from a human patient.

Genome sequencing of *B. bacilliformis* strain INS was carried out at the Peruvian National Institute of Health using the SOLiD 3 Plus genome analyzer, with 700-fold coverage. Sequence reads were aligned to a consensus sequence and were assembled by Bowtie 2 (4) to the *B. bacilliformis* KC583 (NC_008783.1), providing a total of 20 contigs. The protein-coding genes were predicted using Glimmer 3.0 (5); tRNAs and rRNAs were identified with tRNAscan-SE (6) and RNAmmer (7), respectively. The genome sequence was annotated using Rapid Annotations using Subsystem Technology (RAST) (8) and Prokaryotic Genome Annotation Pipeline (PGAAP) (9). The functions of the predicted protein-coding genes were annotated with the NCBI nonredundant (NR) (10) and Clusters of Orthologous Groups (COG) (11) databases. The *B. bacilliformis* INS draft genome sequence has a total of 1,444,107 bp with an average G+C content of 38.3%. It contains 1,244 predicted coding sequences (CDSs), 4 rRNAs, and 44 tRNAs.

Using COG functional assignment, the predicted proteins (73.2%) could be classified into 18 COG categories. There are 950 subsystems represented in the genome. The five most abundant subsystems are related to protein metabolism (*n* = 189); cofactors, vitamins, and pigments (*n* = 122); RNA metabolism (*n* = 114); amino acids (*n* = 99); and DNA metabolism (*n* = 73). In addition, many CDSs are involved in the cell wall and capsule (*n* = 70); membrane transport (*n* = 34); and virulence, disease, and defense (*n* = 32). These findings indicate that strain INS has various genetic processes and a unique adaptation to human blood cells.

Pair-wise BLAST comparisons with Efficient Database framework for comparative Genome Analyses using BLAST score Ratios (EDGAR) (12) of the protein sequences from *B. bacilliformis* INS, *Bartonella henselae* (NC_005956.1), and *Bartonella quintana* (NC_005955.1) revealed 342 CDSs unique to *B. bacilliformis* INS, and the core had 930 genes in common. We identified genes homologous to the invasion-associated locus B family protein, which is responsible for invading human erythrocytes (13).

The INS genome contains genes encoding flagellar proteins, which are essential for erythrocyte invasion (2) and confer an immunogenic effect (14). Other proteins showed similarity to OMP25 and D15 proteins, which have been defined as immunogenic and protective antigens (15–17) and which present mutations in the *gyrA* gene correlated to quinolone resistance (18).

The genomic sequencing of *B. bacilliformis* strain INS will provide insight into the conservation of genomic sequences. The data generated herein will be important for the further understanding of the molecular mechanisms of Carrion’s disease pathology and will generate support for rapid diagnosis by enzyme-linked immunosorbent assay (ELISA), PCR, and possible vaccine targets.

### Nucleotide sequence accession numbers.

This Whole Genome Shotgun project has been deposited at DDBJ/EMBL/GenBank under the accession no. AMQK00000000. The version described in this article is the first version, AMQK01000000.
